# Anticonvulsants in the treatment of aggression in the demented elderly: an update

**DOI:** 10.1186/1745-0179-5-14

**Published:** 2009-06-16

**Authors:** Benedikt Amann, Johannes Pantel, Heinz Grunze, Eduard Vieta, Francesc Colom, Ana Gonzalez-Pinto, Dieter Naber, Harald Hampel

**Affiliations:** 1 Benito Menni, CASM, Research Unit, CIBERSAM, St Boi de Llobregat, Barcelona, Spain; 2 Department of Psychiatry, University of Frankfurt, Germany; 3 Institute of Neuroscience, University of Newcastle upon Tyne, Leazes Wing, Royal Victoria Infirmary, Newcastle upon Tyne, UK; 4 Bipolar Disorders Program, Clinical Institute of Neuroscience, CIBERSAM, University Hospital Clinic, IDIBAPS, University of Barcelona, Barcelona, Spain; 5 Department of Psychiatry, Santiago Apóstol Hospital, CIBERSAM, Vitoria, Spain; 6 Psychiatric Department, University of Hamburg Eppendorf, Germany; 7 Alzheimer Memorial Center, Department of Psychiatry, Ludwig-Maximilian University, Nussbaumstrasse 7, 80336 Munich, Germany; 8 Discipline of Psychiatry, School of Medicine and Trinity College Institute of Neuroscience (TCIN), Trinity College, University of Dublin, Trinity Center for Health Sciences, Tallaght, Dublin 24, Ireland

## Abstract

**Introduction:**

Complex psychopathological and behavioral symptoms, such as delusions and aggression against care providers, are often the primary cause of acute hospital admissions of elderly patients to emergency units and psychiatric departments. This issue resembles an interdisciplinary clinically highly relevant diagnostic and therapeutic challenge across many medical subjects and general practice. At least 50% of the dramatically growing number of patients with dementia exerts aggressive and agitated symptoms during the course of clinical progression, particularly at moderate clinical severity.

**Methods:**

Commonly used rating scales for agitation and aggression are reviewed and discussed. Furthermore, we focus in this article on benefits and limitations of all available data of anticonvulsants published in this specific indication, such as valproate, carbamazepine, oxcarbazepine, lamotrigine, gabapentin and topiramate.

**Results:**

To date, most positive and robust data are available for carbamazepine, however, pharmacokinetic interactions with secondary enzyme induction limit its use. Controlled data of valproate do not seem to support the use in this population. For oxcarbazepine only one controlled but negative trial is available. Positive small series and case reports have been reported for lamotrigine, gabapentin and topiramate.

**Conclusion:**

So far, data of anticonvulsants in demented patients with behavioral disturbances are not convincing. Controlled clinical trials using specific, valid and psychometrically sound instruments of newer anticonvulsants with a better tolerability profile are mandatory to verify whether they can contribute as treatment option in this indication.

## Introduction

A common problem in elderly demented patients is aggressive behavior against nurses or care providers. This is particularly true with a dramatically increasing prevalence and clinical relevance during moderate to severe clinical stages of the disease. Studies conducted in nursing facilities showed that 2/3 of their population suffers from a dementia syndrome and almost all of them demonstrate psychopathological and behavioral symptoms [1-3]. The most common symptoms – normally clinically associated with underlying moderate to severe Alzheimer's disease (AD) – are aggression, irritability and agitation. Half of a sample of outpatients with AD is suggested to present with agitation, 1/3 with violent behavior and 1/4 with verbal outbursts [4].

There still exists a considerable confusion in the psychiatric community as to how aggression and agitation are to be specifically defined and best distinguished [5]. The core features of agitation generally described include restlessness with excessive or semipurposeful motor activity, irritability, heightened responsiveness to internal and external stimuli, and an unstable course [6]. Aggression can be distinguished in a verbal, physical and sexual subtype and is not considered as core feature of agitation. However, features disposing to aggression and the transition from simple agitation to aggression are still poorly understood.

With respect to the etiology of aggression in demented patients Raskind (1999) describes the interaction of three factors, which lead to aggression in this population [5]: The basis is presumably a neurobiological dysregulation, e.g. an hyperdopaminergic state or noradrenergic and γ-aminobutyric acid disturbances in the substantia nigra that may lower the threshold for the expression of aggression. Cognitive impairment in demented patients increases also the aggressive potential due to misperceptions, poor insight or desinhibition. Finally, factors like an unfamiliar environment might lead to an exacerbation of aggressive symptoms. In this respect, climate, noise level and general level of stimulation has to be balanced. If possible at all, changes of these conditions are often not satisfying, even in combination with behavioral therapy or strategies of verbal de-escalation. Therefore, pharmacotherapy plays a significant role in diminishing symptoms, stabilizing mood and consecutively enhancing quality of life for patients, as much as family members and care providers.

## Measures

Aggressive behavior belongs to the complex of so-called non-cognitive symptoms of dementia. These include a variety of psychopathological and behavioral categories including delusions, hallucinations, depression, anxiety, apathy, irritability, euphoria, desinhibition as well as aggression, agitation and disruptive behavior. To classify these neuropsychiatric disturbances the term Behavioral and psychological symptoms of Dementia (BPSD) was coined. The complexity and importance of behavioral and psychopathological phenomena in dementia requires that they can be differentially assessed in the course of the disease and quantified for research purposes [7]. This holds particularly true for the measurement of therapeutic response in drug efficacy studies. However, the lack of clear definition and boundaries between entities creates automatically problems of measurement.

A number of scales have been devised for the assessment of BPSD, but up to now no single scale is considered clearly superior to the others [8]. Nonetheless, there are several significant differences between the available instruments with respect to their validity and psychometric properties. Furthermore, some of these scales are rather unspecific with regard to age and diagnosis, like the Overt Aggression Scale [9], the Overt Agitation Scale [10], and the Brief Agitation Rating Scale (BARS) [11]. Many of the recent studies analysed in this review also apply subitems of the Brief Psychiatric Rating Scale (BPRS/e.g. subfactor hostility or agitation) or very global instruments such as the Clinical Global Impression of Change (CGIC) or even Young-Mania Rating Scale (YMRS) to define treatment response. However, these instruments were not primarily developed for geriatric patients with dementia.

A scale that measures global aspects of BPSD in dementia is the Behavioral Pathology in Alzheimer's Disease Rating Scale (BEHAVE-AD), a 25-item rating scale with assessments on a 4-point severity score [12]. More specific with respect to aggressive and disruptive behavior is the Behavioral and Emotional Activities manifested in Dementia Scale to score troublesome and disruptive behavior with items that have a strong correlation with standard scales like the BPRS and the Sandoz Clinical Assessment-Geriatric [13]. The Troublesome Behavior Scale defines 14 items -in combination with their frequency of occurrence- to describe disruptive or burdensome behavior of the elderly demented patient [14]. Other useful and practicable rating scales are the Cohen Mansfield Agitation Inventory (CMAI), which gives a detailed assessment of agitation [15] and the Disruptive Behavior Rating Scale [16]. The latter includes a set of rating scales used to measure four different dimension of disruptive behavior that present frequent problems in patients with dementia, such as physical and verbal aggression, agitation, and wandering. However, only few experiences exist for the use of the CMAI and the Disruptive Behavior Rating Scale as endpoints in clinical drug trials.

An instrument which is particularly valuable for the use in clinical trial settings is the Neuropsychiatric Inventory (NPI) by Cummings [7,17]. The NPI assesses 12 different subtypes of behavioral disturbances concerning the psychopathology in dementia, including delusions, hallucinations, dysphoria, anxiety, agitation, euphoria, apathy, irritability, aberrant motor behavior, night-time behavior disturbances and change in appetite and eating behavior. It is based on observations made by family or professional caregivers. A screening question is asked first, followed by subquestions if the response to the screening question suggests the presence of abnormalities involving that neuropsychiatric domain. After administration of the subquestions, the caregiver rates the frequency and severity of each abnormality as well as the caregivers distress associated with each neuropsychiatric alteration. Having two different scores for severity and frequency for each domain potentially increases the utility of the NPI in drug efficacy studies because effects on frequency (events occur less frequently but each event has not changed severity) versus effects on severity (events occur just as often after treatment but are of diminished intensity) can be separated. Previous studies suggest that the NPI is psychometrically sound, comprehensive and sensitive to change [17]. Accordingly, it has been successfully used in drug trials to score changes in disruptive behavior and other neuropsychiatric symptoms after treatment [18-21].

Many of the more recent studies analysed in the present review do not use specific scales for the assessment of BPSD but rather apply instruments which were originally developed for general adult psychiatric populations (e.g. BPRS or the CGIC). This relates to the fact that many of the more detailed and specific instruments for the valid assessment of BPSD became only available during the last decade. This does not necessarily imply that the results are less meaningful but should be taken in mind as a potential limitation when interpreting the conclusions of those older studies.

## Anticonvulsants

Anticonvulsants, such as carbamazepine (CBZ), valproate (VPA) and lamotrigine (LTG) have become established treatment options in bipolar disorder. In this article we will update their potential effectiveness as antiaggressive drugs in elderly demented patients, looking also into newer drugs of this class like oxcarbazepine (OXC), topiramate (TOP) and gabapentin (GPN).

## Carbamazepine

CBZ shows a blockade of voltage-dependent sodium, and, additionally, calcium L- type channels. Furthermore, CBZ reinforces repolarization by potassium outflow, and has GABAergic, adenosinergic and glutamate antagonistic properties. In the kindling model that serves as a lab bench model for the progression of epilepsies and recurrent affective disorders, CBZ shows reasonable antikindling properties [22]. CBZ's efficacy in behavioral syndromes might be related to its inhibitory effects on limbic system kindling, increased locus coeruleus firing and enhanced tryptophan levels [23].

First reports of CBZ in the treatment of emotionally disturbed patients are reaching back to the 70 s [24,25]. In the following decade some single case reports of CBZ followed showing improvements in behavioral disturbances in demented patients [26-28].

CBZ was also administered in an open trial to eight patients with organic brain disease characterized by aggressive and assaultive behavior refractory to conventional treatment [29]. Improvement was observed in all patients, with the average number of assaults over pre-treatment and post-treatment observation periods declining by more than half. Nine outpatients who met well-defined criteria for probable AD and who had significant behavioral agitation failed to improve with antipsychotic therapy and were subsequently treated with CBZ in another open study [30]. Five patients showed a clear improvement, and one patient an equivocal response according to clinical evaluation and BPRS scores.

A further open prospective study investigated the effects of CBZ on agitation, hostility, and uncooperativeness in 15 severely demented Alzheimer's inpatients who had failed to respond to prior treatment with antipsychotics [31]. Severity of psychopathologic symptoms was again assessed by the BPRS with a significant improvement in activation and hostility after 4 weeks.

A negative small placebo-controlled, double-blind study of CBZ in 19 elderly demented patients was reported in 1982 [32]. In dosages of 100–300 mg/d for a period of 4 weeks no response was noted in wandering, aggression or agitation and a worsening of cognitive functioning was even observed using the Behavior Rating Scale and the Clifton Assessment Scedule (CAS). Low doses (average serum levels 3.5 microg/ml) of CBZ might be a possible reason for these negative results.

More promising results evolved in two further controlled studies by Tarot and colleagues [33,34]. The first was a nonrandomized, placebo-controlled, crossover trial with 25 patients with dementia and verbal or physical aggression [33]. Median total BPRS score decreased 7 points on CBZ versus 3 on placebo. Sixteen subjects were rated as improved globally on CBZ versus four on placebo. CBZ was titrated to serum levels between 5 and 8 microg/ml with good tolerability. The following 6-week, randomized, multisite, parallel-group study, screened 163 patients and finally included 51 severely demented patients who received up to 300 mg CBZ or placebo for 6 weeks [34]. CBZ was started with 100 mg/d and increased -according to the protocol- by 50 mg every 2–4 days. If no toxic side effect occurred, a serum level of 5–8 microg/ml was maintained. CGIC, BPRS, Overt Aggression Scale and Behavior Rating scale of dementia were applied. Over 6 weeks the mean total BPRS score decreased 7.7 points for the CBZ group and 0.9 for the placebo group, and the weekly scores indicated a gradual divergence between the two groups. CGI ratings showed global improvement in 77% of the patients taking CBZ and 21% of those taking placebo. Side effects did not occur significantly more often in the CBZ group compared to placebo group. Therefore, serum levels of 5–8 microg/ml were recommended by the investigators. One patient was withdrawn from the study because of tics and extensive sedation occurring at a dose of 200 mg/d. One patient developed ataxia, but remained in the study. No deterioration of cognitive function was observed.

Another 6-week, randomized, double-blind, placebo-controlled, parallel-group trial investigated 400 mg CBZ in 21 agitated subjects (16 completers) who had been treated unsuccessfully with antipsychotics. A greater improvement in the CBZ group than in the placebo group on the CGI and especially on the hostility item of the BPRS was observed [35].

In conclusion, CBZ seems to be an effective treatment in a reasonable number of severely demented and agitated patients. However, the efficacy and safety of long-term use remain to be established and use is often limited by pharmacokinetic drug interactions secondary to its enzyme-inducing effect.

## Oxcarbazepine

Oxcarbazepine (OXC) is the keto-derivative of CBZ and represents an alternative to CBZ due to its suggested better tolerability profile. In a randomized, placebo-controlled, double-blind trial in patients with bipolar disorder a significant effect of this drug in the prevention of impulsivity and related behaviors was observed [36], suggesting that it might also play a role for the treatment of impulsivity in patients with dementia. So far, only one clinical study in this population has yet been published to investigate this hypothesis. To evaluate the efficacy of OXC in the treatment of agitation and aggression in patients with AD, vascular dementia or both a pharmaceutical industry independent 8-week, multicenter, randomized, double-blind, placebo-controlled trial was published recently [19]. Changes in the agitation and aggression subscore of the NPI were the primary outcomes. The secondary outcomes were the changes in the caregivers' total burden scores (measured by the NPI) and changes in the Brief Agitation Rating Scale. In total, 103 institutionalized patients at 35 sites were randomized to the trial. After 8 weeks, no statistically significant differences were found between the 2 groups for all outcomes. A trend was observed in favour of the OXC group in the reduction in the scores on the BARS.

Due to this negative controlled study, at present the use of OXC in this indication can not be recommended. Results, however, have to be replicated but its use in dementia might be limited as OXC causes more often hyponatremia than CBZ, especially in older patients [37,38].

## Valproate

VPA blocks voltage-dependent sodium channels and calcium T- type channels and reinforces repolarisation by shifting the inactivation curve of early potassium outward currents towards more positive membrane potentials. Furthermore, it has indirect GABAergic and serotonergic properties. On the intracellular level, it interferes with the inositol phosphate metabolism and activates antioxidant cell survival proteins, e.g. bcl-2 or Rho-Kinases [39]. In the kindling model VPA has pronounced antikindling properties [22].

VPA has a variety of indications. Besides epilepsy and acute mania, it seems to be effective in panic disorder, alcohol and sedative withdrawal, agitation as well as behavioral dyscontrol [40,41]. In an open trial 10 patients diagnosed with personality disorder with impulsive aggressive behavior were treated with VPA [42]. In six of 8 completers irritability and impulsive aggressive behavior improved significantly.

One of the first open trials with VPA in the treatment of severe behavioral disturbances in dementia was conducted in 4 patients receiving VPA for 1 to 3 months [43]. Two patients showed significant improvement in behavior, a third had a transient response. Another open trial in elderly demented and agitated patients was conducted in 1995 [44]. A global rating scale was used by the nursing staff biweekly to assess improvement which was a 50% or greater reduction in the frequency of episodes of behavioral agitation in 8 of 10 patients. The dosage was 375–750 mg/d and was well tolerated and safe. Similar positive results were documented by Haas and colleagues (1997) in a trial with 12 cognitively impaired and aggressive elderly patients and by Porsteinsson and colleagues (1997) who reported on 13 patients suffering from agitation and neuropsychiatric disorders [45,46]. Drug response was rated on the CGIC.

VPA alone or in combination with antipsychotics was compared in an open study with 25 patients suffering from dementia with behavioral disturbances [47]. Using also the CGIC a total of 56% of all included subjects responded with 7 out of 15 patients (47%) when given VPA in monotherapy and with 7 out of 10 (70%) when added to antipsychotic treatment. The mean daily dose was 1650 mg/d with a mean blood level of 64 microg/ml. Except for reversible sedation in 8 patients and transient worsening of gait and confusion in 1 subject, no other side effects were observed. In an open-label extension trial of a double-blind study ongoing treatment with VPA (mean: 851 mg/d) was associated with improvement in measures of agitation in 46 patients [48]. Behavior was rated on the Social Dysfunction and Aggression Scale 9 (SDAS-9), a Dutch version of the Behavioral Observation Scales for Intrmural Psychogeriatrics (GIP), and the CGIC. Sixty percent of subjects had no side effects and 33% had side effects that were rated as mild.

The goal of a study by Forester and colleagues (2007) was to determine if manifestations of agitation, such as physical aggression, physically nonaggressive or verbally agitated behavior, show different degrees of response to VPA alone or in combination with second-generation antipsychotic agents [20]. In a 6-week, open-label, naturalistic pilot study of patients aged > 60 years recruited from a geriatric psychiatry inpatient unit, 2 nursing homes, and 4 assisted living residences the primary outcome measure was measured on the CMAI and the NPI -Nursing Home version (NPI-NH). Fifteen patients were included in the study. Patients with higher levels of agitation receiving VPA had reduced agitation on the physical aggression subscale of the CMAI. VPA was less effective on physically nonaggressive behavior and verbal agitation. Irritability, as measured on the NPI-NH, was also reduced. Patients who received both VPA and an antipsychotic agent were responsive at lower doses of VPA. In either case, the effective dosage of VPA was lower than that commonly used for epilepsy or mania in elderly patients. The most common adverse events included somnolence and gait disturbance.

The positive results of open-trials are somewhat in contradiction with results of controlled trials of VPA in demented patients with behavioral disturbances.

In 2002 a randomized, placebo-controlled, double-blind cross-over study was published with 42 demented and aggressive patients treated with a fixed dose of 480 mg/d of VPA (plasma levels of VPA: 40,9 +/- 10,8 microg/ml) [49]. Primary outcomes were again the SDAS-9 and the CGIC. The treatment with VPA showed no difference compared to placebo on aggressive behavior but significant improvement on restless, melancholic and anxious behavior. Possible limitations of this study were the low dose of VPA and the relatively short treatment period. 39 patients were further examined in a twelve-week open label follow-up study [50]. Maintaining the same dosage of VPA aggressive, non-social, apathetic, disorientated behavior and distorted memory improved at week 12 compared to week 0. The authors used apart from the SDAS-9 the Behavior Observation scale for Intramural Psychogeriatrics and the CGI.

A large placebo-controlled study included 153 nursing home residents with probable or possible AD complicated by agitation [51]. Patients receiving a mean dose of 800 mg/d VPA over a period of 6 weeks did not differ from patients treated with placebo. The primary outcome measure was change from baseline on the BPRS Agitation factor. Secondary outcomes included total BPRS, CGIC, CMAI score, and measures of safety and tolerability. In summary, this study did not support the use of VPA in demented elderly and agitated patients.

Another very small randomized, double-blind trial was conducted with 14 patients with AD and agitation [21]. Patients were assessed with the NPI and CMAI at baseline and after 6 weeks of treatment with VPA and placebo, with 2 weeks between phases to allow for placebo washout and tapering. The results were also negative and agitation and aggression scores even worsened during VPA treatment when compared to placebo.

In conclusion, it seems that VPA currently can not be recommended in demented and aggressive elderly patients, even though open trials and extension studies of controlled trials are positive and some of the controlled studies -as stated before- show some limitations, such as small numbers of patients included or low doses of VPA. A Cochrane review published in 2004 concluded that VPA in low doses is ineffective to control agitation/aggression in demented patients and that higher doses of VPA are associated with unacceptable side effects [52].

## Lamotrigine

Lamotrigine (LTG) became a main focus of interest beyond its primary indication, epilepsies, as it is sharing many cellular mechanisms of action with the established mood stabilizers CBZ and VPA. It mainly reinforces the repolarisation by enhancing an early potassium outward current [53], blocks voltage-dependent sodium and calcium channels and reduces glutamate release. Intracellular actions comparable to VPA may be assumed, but have not been elucidated so far. In the kindling model LTG has similar pronounced antikindling properties as VPA [22]. Furthermore, LTG appears to have neuroprotective effects but its potential to induce severe cutaneous side effects has to be taken into account.

One case report and one case series indicate that LTG might be a successful strategy in patients with dementia and aggressive behavior or agitation [54,55]. The first publication reports on a dramatic improvement with the introduction of LTG (100 mg/d) in a patient with frontal lobe dementia and a large history of verbal and physical aggressions [54]. A retrospective medical record review of the effectiveness of LTG for manic-like symptoms and agitation in 5 demented patients was conducted in the second [55]. In dosages of LTG between 100 and 300 mg/d patients showed a decrease in the Young-Mania Rating Scale through 5 months. Obvious limitations of this case series are the small number of patients and the use of a scale, which is normally applied in bipolar disorder.

To define the role of LTG in this indication, larger and controlled trials are warranted.

## Gabapentin

Gabapentin, with indirect GABAergic properties, may act mainly via blockade of L type calcium channels and possibly sodium channels as well; however, its decisive mechanisms of action both in epilepsy and pain treatment are still unclear.

In a case report of a patient suffering from vascular dementia and aggressive behavior the addition of gabapentin resulted in reduced agitation, sexual inappropriateness, and lability [56]. The dosage of 900 mg/d was well tolerated. An open 15-month investigation of gabapentin included 20 demented patients with behavioral alterations [57]. The results of this study in patients with probable AD with behavioral alterations and serious comorbidities indicate that gabapentin provides significant and sustained efficacy in terms of behavior, with associated reductions in caregiver burden. No patient withdrew during the study and no side effects or drug interaction occurred. A recent review of gabapentin in this indication suggests benefits but a limited support for the off-label use of gabapentin for the treatment of BPSD due to the dearth of available data [58].

In conclusion, gabapentin in aggressive demented patients seems to be worthwhile to be investigated further, also due to its good tolerability profile, but controlled studies are mandatory to verify its role in BPSD.

## Topiramate

Topiramate (TOP) is a structurally unique antiepileptic drug in so far as it belongs to a sulfamate-substituted d-fructose substance group. However, many of the known mechanisms of action of TOP are similar to those of established AEDs, especially the putative mood stabilizers CBZ, VPA and LTG. TOP modulates sodium conductance, inhibits L-type calcium channels at low serum concentrations, potentiates GABAergic inhibition, decreases AMPA/kainate receptor-mediated currents and is a weak inhibitor of carbonic anhydrase [59].

TOP has a good tolerability profile but a possible cognitive impairment has to be taken into account when applied to demented patients [60].

Fhager and colleagues (2003) retrospectively evaluated the outcome of TOP used on 15 demented patients who were severely aggressive and failed to respond to antipsychotics [61]. Patients received between 25 mg to 150 mg TOP daily either in monotherapy or additional to an antipsychotic. Symptoms were rated using Cohen Mansfield Agitation Inventory at baseline and 2 weeks of receiving TOP. Both groups showed significant improvement in aggressive behavior and no side effects were reported. Despite of these positive results its use in demented patients has to be considered with caution, because of its lack of controlled data. Furthermore, a possible deleterious effect on cognitive functions in this fragile population has to be considered.

## Conclusion

With an increased life expectancy the global population will age and suffer more frequently from dementia and associated non-cognitive problems, such as aggressive or agitated behavior. A recent prevalence study of BPSD in dementia confirmed again that nearly all 587 demented subjects suffered from behavioral and psychological symptoms [62]. Assessment and treatment of dementia therefore is crucial and important to reduce stress in affected patients and their families. An individual treatment plan with cognitive behavioral therapeutic approaches and verbal de-escalation might be the best option to cope with demented elderly and aggressive patients. Its application, however, remains often unrealistic due to a lack of financial resources to enhance education and the presence of nurses or care providers in nursing homes. Therefore, pharmaceutical interventions will often remain necessary. Unfortunately, emerging evidence indicates that some treatment options, such as typical and atypical antipsychotics, have limitations in this population due to possible side effects, such as extrapyramidal, cardiovascular or metabolic side effects.

Anticonvulsants are an interesting class for the potential use in this population as they are successfully prescribed in similar behavioral disturbances and in bipolar disorder. Three reviews on the use of anticonvulsants in behavioral alterations in demented patients were published in the last three years [63-65]. Whereas Guay (2007) was more supportive on the use of anticonvulsants in this indication [63], the other two authors were rather critical and pronounced the lack of controlled data [64,65]. In our opinion, the data of anticonvulsants in demented patients indeed are not convincing, even though some patients clearly benefit from the use of anticonvulsants. Most data are available for CBZ and VPA so far. CBZ seems to be the most effective drug, but pharmacokinetic interactions and side effects are likely to occur, particularly in this vulnerable population which is often treated with polypharmacy. VPA is better tolerated but controlled data are not conclusive for the use in this indication. The literature regarding newer anticonvulsants, such as OXC, LTG, gabapentin or TOP, is modest in volume. With one negative controlled study OXC can not be recommended so far, but certainly more studies are warranted to possibly replicate findings. Controlled trials in future with LTG, gabapentin or TOP will help us to understand its effectiveness and safety in demented patients with behavioral alterations. However, possible side effects, such as hyponatremia with OXC or cognitive impairment with TOP have to be taken into account when new studies are planned.

One limitation of a majority of the published studies is the use of rather global and non-specific instruments for the measurement of drug response. This applies particularly for the more recent studies. In the meantime several more specific, valid and psychometrically sound instruments for the assessment of behavioral disturbances in dementia (e.g. the NPI) are available and evaluated with respect their usefulness and validity. These should be applied in future studies.

## Competing interests

Dr. Vieta has received grants and acted as consultant for Astra-Zeneca, BMS, Forest, GSK, Jazz, Janssen, Lilly, Novartis, Pfizer, Otsuka, Sanofi-Aventis, Shering-Plough, and Servier.

Heinz Grunze received grants and/or research support, consulting fees and honoraria within the last three years from Astra Zeneca, Bial, BMS, Eli Lilly, Glaxo-Smith Kline, Janssen-Cilag, Organon, Pfizer Inc, Sanofi-Aventis, Servier, UBC and UCB Belgium.

Benedikt Amann, Harald Hampel and Johannes Pantel report no competing interests with the issues and pharmacological compounds discussed in this article.

## Authors' contributions

BA had the idea and wrote the first draft of the manuscript. EV, AGP, HH, JP and HG revised, modified and corrected the manuscript, whereas FC and DN added helpful comments to improve the quality of the paper. Finally, HH and JP helped to revise the manuscript after first submission. All authors read and approved the final manuscript.
